# Gossypiboma: the failure of a successful intervention

**DOI:** 10.11604/pamj.2020.36.335.25464

**Published:** 2020-08-25

**Authors:** Atef Mejri, Khaoula Arfaoui, Badreddine Aloui, Jasser Yaakoubi

**Affiliations:** 1 General Surgery Department, Regional Hospital of Jendouba, Jendouba, Tunisia

**Keywords:** Surgery, complication, iatrogenia, textiloma, gastrointestinal perforation

## Abstract

If successful surgery is the primary quest of any surgeon, unintentionally leaving behind surgical items in the operative field remains his most feared obsession. This rare but dramatic accident can lead to potentially fatal complications and turn both lives of the surgeon and the patient upside down. We present the case of a 29-year-old female patient who presented to the ER with three days history of severe diffuse abdominal pain associated with fever, biological inflammatory syndrome and well-tolerated iron deficiency anaemia. She had no past medical history except for a lower segment cesarean section 5 months ago. Abdominal MRI allowed the diagnosis of two gossypibomas responsible for two intra-abdominal collections. An emergency laparotomy allowed the removal of these foreign bodies and the management of their serious complications of intestinal perforation by the construction of a double intestinal stoma. The patient made a post-operative uneventful recovery. This observation emphasizes the need to raise the practitioner´s awareness about this differential diagnosis in every case of any poorly localized abdominal pain occurring after surgery.

## Introduction

The term gossypiboma refers to any type of non-absorbable surgical swab mistakenly left inside the human body after surgery. It concerns primarily the abdominal cavity (56%) then the pelvis (18%) [1]. This iatrogenic condition may have unfortunate health consequences with severe morbidity for the patient. Its real incidence is difficult to estimate given its medico-legal [2] implications and it is reported to occur in 1/1000 to 1/1500 intra-abdominal procedures. The diagnostic delay added to the severity of the possible complications may jeopardize the vital prognosis.

## Patient and observation

A 29-year-old female patient presented to the emergency room with a several month history of diffuse abdominal pain associated with episodes of diarrhea nausea and chills. Further investigations revealed a history of cesarean section via pfununstiel incision for fetal macrosomia 5 months ago. There was no history of any other previous disease or surgery. The patient claimed that few weeks following her delivery; she started to suffer from dull abdominal pain, vomiting and diarrhea. Her general physician attributed her symptoms to a puerperal infection and she was sent back home with an array of antibiotics and painkillers. Starting one month before admission, she had multiple episodes of melena totally neglected by the patient. On physical examination, her body temperature was 38.9°C. She had a blood pressure of 110/80 mm/Hg, a heart rate of 88 beats/min and respiratory rate of 16 breaths/ min. The abdomen was slightly distend and tender especially in the right and left iliac fossa without guarding.

**Figure 1:**
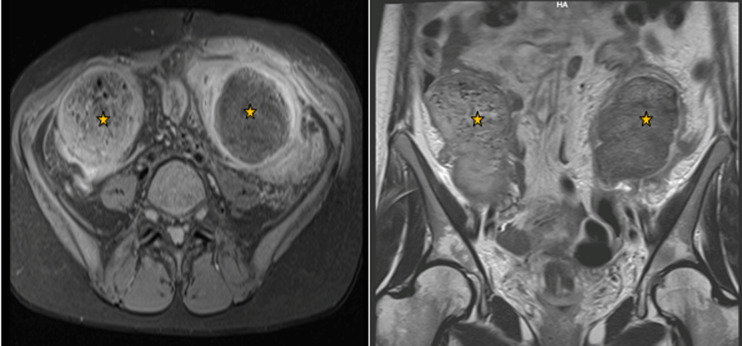
MRI views showing two well-circumscribed collections located in the right and left iliac fossa with spongiform pattern and entrapped air bubbles

**Figure 2:**
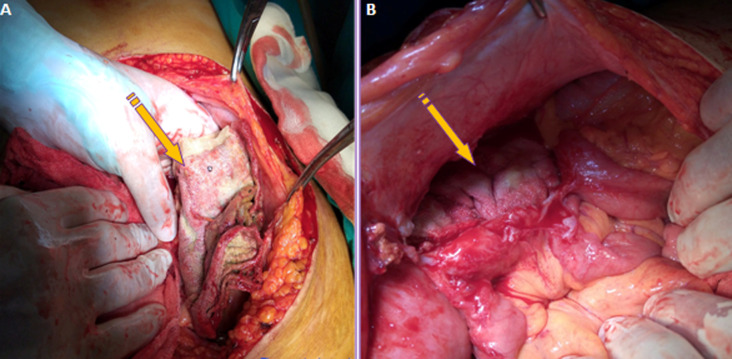
A) intra-operative view showing the surgical towel found in the perisigmoid collection; B) the surgical towel found in the right iliac fossa

**Figure 3:**
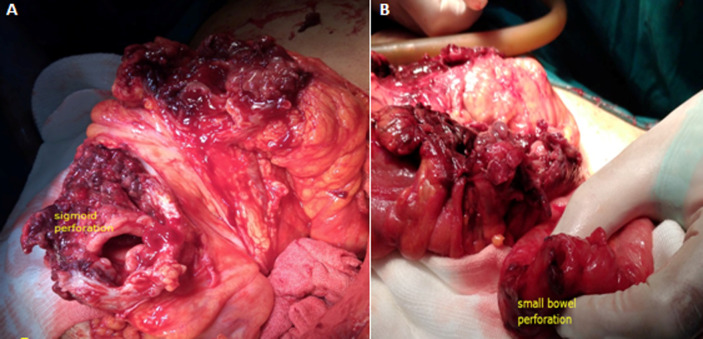
A) intra-operative view showing the sigmoid colon perforation; B) the small bowel perforation

Bi-digital examination did not find any abnormalities. Initial screening tests showed a biological inflammatory syndrome with a WBC count of 21,000 cells/mm^3^ and a CRP rate of 180mg/l. It also showed an iron deficiency anaemia with a hemoglobin value of 9g/dl and a mean corpuscular volume of 69fL. Given the unavailability of the CT scan that day for technical reasons, an emergency abdominal MRI showed two well-circumscribed collections located in the right and left iliac fossa with spongiform pattern and entrapped air bubbles (Figure 1). The patient underwent emergency surgery using a midline incision. A thorough examination found a per sigmoid collection containing a surgical towel (Figure 2) which became adhered to the sigmoid colon and caused its perforation (Figure 3) and another collection in the right iliac fossa containing another surgical towel (Figure 2) having caused a small bowel perforation located 70 cm from the ileo-caecal valve (Figure 3). After the removal of the two retained textile foreign bodies and a peritoneal lavage, the small bowel perforation justified an ileostomy. A sigmoidectomy with a Hartmann precedure was also performed with good outcome. The post-operative period was uneventful. The patient was discharged on day 9 after surgery in good condition. During the medical follow-up visits, she reported a post-traumatic stress disorder and depressed mood that required psychological treatment. Restoration of colorectal continuity is scheduled three months after surgery.

## Discussion

Forgetting surgical material of cotton matrix in the operating field is most commonly described in emergency surgical procedures, those with prolonged operative duration and hemorrhagic interventions [3]. High body mass index has also been cited as another contributing factor [1]. Literature data corroborates our observation indicating that cesarean section alone exposes to this risk with an incidence of 17.9% [4]. Physiopathologically, the retained sponges or gauze left by mistake in the peritoneal cavity can induce an exudative inflammatory reaction. In case of secondary infection, these surgical materials and the surrounded foreign body reaction may turn into a real intra-abdominal abscess. It can also encyst in adherent tissue and become calcified. Aseptic fibrosis may in some cases give rise to an encapsulated mass that remains silent for years [3]. Sankpal et al. have described a case of a completely accidental discovery of a textiloma during a laparoscopic cholecystectomy following a cesarean section occurring 5 years earlier [3]. Longer delays in finding a textiloma after a cesarean section have been reported in literature. Abdulrehamn et al. reported another case of a textiloma discovered up to 15 years after a classical caesarean delivery [5].

The clinical presentation lacks specificity and outside the framework of accidental discovery, the signs generally reflect a complication such as abscess, intra-abdominal collections, acute intestinal obstruction, acute peritonitis due to intestinal perforation or internal or external fistulas [6]. Chief complain of the patient is persistent abdominal pain. Clinical symptoms may include abdominal tenderness or guarding in case of peritonitis, fever, gastro intestinal disorders, alteration of the general condition, bleeding and a palpable abdominal mass [1]. The case presented in our observation highlights the ability of these foreign bodies to cause progressive erosion through the intestinal wall leading to perforation. In such a case, routine blood investigations may show a biological inflammatory syndrome. Trans-mural migration can mimic a pseudo-tumour responsible for gastro intestinal disorders, melena and anaemia, as it is the case in our patient. Hence, the danger of this incident, which could completely change the outcome of the simplest commonly performed surgical intervention and worsen its prognosis. It should be noted however, a case of complete trans-mural migration of a spontaneously expelled textiloma without damage by natural rectal route described by Butt et al. [7].

The clinical sings are strongly helped by the radiological findings in order to establish a timely diagnosis [4]. The plain abdomen X-ray may be sufficient to detect the radiopaque marker if it is present on the textile used but with a false negative rate of 25% [4]. Abdominal CT is the best imaging modality that allows making the diagnosis, indicating the exact location of gossypiboma and precising its possible complications. It usually shows a highly suggestive typical image of a heterogeneous spongiform cystic lesion with a hyperdense line indicating the radio opaque ribbon [6]. A calcified “reticulate rind sign” may also be noticed in cases of long-lasting gossypiboma and represents a very useful characteristic radiological feature [8]. MRI is another diagnostic alternative and it shows a well-defined hypointense cystic lesion on T1 weighed images and hyperintense on T2 weighed images. However, it does not visualise the radiopaque marker usually lacking radio-magnetic characteristics and giving poor MRI signal [2].

Consequently, the diagnosis seems difficult and it requires the contribution of multiple associated arguments. The history of a previous surgery, the type of the intervention performed, the biological inflammatory syndrome if present and above all the often very suggestive radiological data are the pillars of the diagnostic process. Once the diagnosis is made, the immediate removal of any gossypiboma associated with high specific level of intensive critical care is necessary to prevent more serious complications that could jeopardize the patient's life. Laparoscopy can be useful in some uncomplicated cases [3]. However, in the event of multiple digestive perforations, as in our case scenario, that can go unnoticed in laparoscopy, laparotomy seems a more reasonable choice. Admittedly, the best treatment remains prevention. Strict compliance with the rules of standard material counting protocols, coordination between members of successive surgical teams, the use of radio opaque marked surgical sponges, the use of modern means of retained sponge detection such as radio frequency or barcode technology help to rectify this human error and avoid its disastrous consequences [6].

## Conclusion

Despite the evolution of the health care system and the continuous modernization of the surgical techniques, gossypiboma is an iatrogenic complication that is still seen in every day´s surgical practice. The diagnosis is difficult because we refuse to admit it and even think about it given its implications for the surgeon´s reputation that mark his professional career for life. Nonetheless, human error still exists and it must be kept in mind justifying a timely radiological examination. Prompt surgical management is essential because any delay worsens the consequences for the patient and the surgeon. Increased vigilance must be shown over preventive measures and strategies aiming to improve safety that must be the subject of constant research.

## References

[1] Bilali V, Bilali S, Mitrushi A, Pirushi R, Nina H, Ktona E (2019). Gossypiboma in abdomen: retained surgical gauze after a cesarean section. G Chir.

[2] Mathew RP, Thomas B, Basti RS, uresh BH (2017). Gossypibomas, a surgeon´s nightmare patient demographics, risk factors, imaging and how we can prevent it. Br J Radiol.

[3] Sankpal J, Tayade M, Rathore J, Parikh A, Gadekar D, Fathima SS (2020). Oh, My Gauze!!!-A rare case report of laparoscopic removal of an incidentally discovered gossypiboma during laparoscopic cholecystectomy. International Journal of Surgery Case Reports.

[4] Gavrić Lovrec V, Cokan A, Lukman L, Arko D, Takač I (2018). Retained surgical needle and gauze after cesarean section and adnexectomy: a case report and literature review. J Int Med Res.

[5] Rehman A, Baloch NU-A, Awais M (2014). Gossypiboma diagnosed fifteen years after a cesarean section: A case report. Qatar Med J.

[6] Rabie ME, Hosni MH, Al Safty A, Jarallah AM, Ghaleb HF (2016). Gossypiboma revisited: A never ending issue. International Journal of Surgery Case Reports.

[7] Butt UI, Shafiq AB, Umar M, Ashfaq M, Ayyaz M (2018). Transmigration and spontaneous passage of a gossypiboma documented on contrast study. Ann Med Surg (Lond).

[8] Lu Y-Y, Cheung Y-C, Ko S-F, Shu-Hang Ng (2005). Calcified reticulate rind sign: A characteristic feature of gossypiboma on computed tomography. World J Gastroenterol.

